# The Anatomical Remembrancer

**Published:** 1846-05-01

**Authors:** 


*The Anatomical Remembrancer, or complete Pocket Anatomist, &c., from the second London edition revised: Published by* Samuel S. *& William Wood, New York.*

This is a duodecimo of 245 pages, and contains a concise description of the bones, ligaments, muscles, viscera; distribution of nerves, blood-vessels and absorbents; arrangement of the several fasciæ; organs of generation, male and female, and the organs of the senses. The usefulness of such a volume to the medical student, especially during dissections, to the practical surgeon at all times, and, scarcely less so to the physician, is too obvious to require amplification. Anatomy lies at the foundation of medical and surgical science, and the practitioner of some years standing, cannot but have learned, by experience, the necessity of frequently refreshing his memory with anatomical details, if he would have them ready at his command when it is important to recal them for practical application. The "Remembrancer" is adapted to this object, and every medical man, as well as pupil, would do well to have it on his table.

